# Added sugar intake among the saudi population

**DOI:** 10.1371/journal.pone.0291136

**Published:** 2023-09-08

**Authors:** Noara Alhusseini, Majed Ramadan, Salwa Aljarayhi, Waad Arnous, Mohamed Abdelaal, Hala Dababo, Bana Dalati, Ola Al Doumani, Sara AlNasser, Rimah Saleem

**Affiliations:** 1 College of Medicine, Alfaisal University, Riyadh, Saudi Arabia; 2 King Abdullah International Medical Research Centre, Riyadh, Saudi Arabia, King Saud Bin Abdulaziz University for Health Sciences, Riyadh, Saudi Arabia; 3 Communication and Swallowing Disorders Division, Prince Sultan Military Medical City, Riyadh, Saudi Arabia; Zagazig University Faculty of Agriculture, EGYPT

## Abstract

Diet is a contributor to the pathogenesis of many non-communicable diseases. Among contributors to poor diet is high added sugar consumption, which is unfortunately on the rise nowadays. The recommended sugar intake by The American Heart Association (AHA) is 24g/day and 36g/day for women and men, respectively. The study’s aim is to assess added sugar intake among adults in Saudi Arabia. A cross-sectional study design was used via an online survey among adults in Saudi Arabia using convenience sampling, and social media platforms were used to collect the data. The authors conducted descriptive statistics to present demographic variables using Chi-square χ2 tests for categorical and t-tests for continuous variables. All statistical tests used a 95% confidence interval with a two-sided P-value <0.05 as significance level. A total of 1163 respondents were included in the study. The study has shown an overall added sugar intake average of 73 g/day. There was a significant difference in means of overall added sugar intake across genders for the age group 18–30 and the age group >60. Equivalently, there was a statistically significant difference in means of added sugar intake food across gender (P-value 0.008). Females tended to consume more added sugar in their food than males. The highest consumption was in the Northern region (123.71 g/day), followed by the Southern region (98.52 g/day), the Western region (86.14 g/day), and lastly, the Central and Eastern regions (66.95 and 62.02 g/day, respectively). The total added sugar intake of added sugar is extremely high in Saudi Arabia. Poor dietary habits lead to many adverse health consequences, including obesity and diabetes. Healthcare providers and public health officials are highly encouraged to shed light on added sugar consumption and create opportunities to promote healthy dietary patterns. The Saudi population is recommended to abide by the added sugar dietary recommendations to avoid future chronic medical conditions.

## Introduction

Sugar is a carbohydrate included in daily diets. Simple sugars are derived from single units called monosaccharides like glucose, fructose, and galactose. When two monosaccharides attach, a disaccharide is formed, such as sucrose, maltose, and lactose. The common table sugar (sucrose) used is made up of glucose and fructose and is extracted from sugar cane or sugar beet [1]. Natural sugars are found in unprocessed foods like fruits and dairy products. Moreover, foods containing natural sugars fruits and honey have health benefits like fibers and antioxidants. On the other hand, added or refined sugars are added while processing packaged foods, like cereal, chocolates, candy, and soft drinks, and do not provide any nutrients besides calories [2].

A significant contributor to a poor diet is high sugar consumption, especially added sugars. An unhealthy diet contributes to the pathogenesis of many diseases, such as obesity, diabetes, cancers, and heart diseases. Non-communicable diseases are the leading cause of death nowadays responsible for 68% of the world’s deaths in 2012. Obesity predisposes to many illnesses, including metabolic syndrome diseases, dyslipidemia, elevated blood pressure, diabetes, non-alcoholic fatty liver disease, cognitive decline, and cancer [3]. Therefore, governments and health organizations have recommended limiting sugar intake to minimal amounts of total energy requirements [4].

Over the past few decades, Saudi Arabia has gone through significant economic progress, and this success and prosperity impacted people’s lifestyles drastically. Ultimately, Saudis started adopting a sedentary lifestyle and an unhealthy dietary pattern, such as increased consumption of fast foods and sugar-dense beverages [5]. Simultaneously, technological advances—including reliance on cars, elevators, escalators, remotes, and food applications- have led to decreased physical activity. Traditional dependence on locally grown natural products such as fruits, vegetables, and wheat has also shifted toward reliance on imported and processed foods [6]. The new shift in lifestyle and diet has resulted in a dramatic increase in diabetes prevalence among adults in Saudi Arabia, as recent studies estimated a prevalence of 17.7% and 16.4% in males and females, respectively [7], and obesity prevalence of 24.7%. Therefore, diabetes and obesity are serious public health problems in Saudi Arabia, as approximately one out of five Saudis is diabetic, and one out of four is obese [8].

International Dietary guidelines vary regarding the recommended daily amounts of added sugar intake. The World Health Organization (WHO) dietary guidelines recommend limiting added sugar intake to less than 10% of daily energy intake [9]. For instance, for a 2000-calorie daily diet, the allowed daily value of added sugars is 200 calories or 50 grams per day. In the United Kingdome (UK), the Scientific Advisory Committee on Nutrition (SACN) recommends that free sugar intake not exceed 5% of total energy intake [10].

Many individuals consume above the recommended sugar levels, impacting their health. Sugar consumption can be addictive. Individuals crave and binge on free sugar, as it gives them a rush of dopamine and serotonin (neurotransmitters responsible for pleasure and satisfaction) [11]. However, the dopamine spike does not last long as sugar is quickly absorbed and metabolized. Therefore, satisfaction subsides quickly, and craving returns shortly. Over time, the same amount of sugar will not provide the dopamine surge as before, and the only way to restore that pleasure is by consuming a higher amount of sugar more frequently [1]. In conclusion, high sugar consumption is a significant issue among many populations, including the Saudi people. As a result, more light should be cast on rising sugar intake, and demographic studies should be conducted to develop applicable regulations and guidelines.

## Materials and methods

This study is a cross-sectional study via convenience sampling. The authors used an online survey to collect the data. The online survey was conducted using the Google Forms web survey platform in English and Arabic. The link to the online survey questionnaire was distributed through social media platforms such as Twitter, WhatsApp, Telegram, LinkedIn, and Facebook.

The inclusion criteria for the study were: (1) adults over 18 years and (2) residing in Saudi Arabia, excluding children and individuals not residing in Saudi Arabia. Informed consent was obtained from all participants for inclusion in the study.

Sample size: Based on our non-probability convenient sampling technique, we utilized the population proportion and size, which was estimated to be 38 million, to determine our minimum required sample size. Using SAS 9.4 software, we calculated the sample size needed with a 95% confidence level, 5% margin error, and 80% power. Through these calculations, we determined that a minimum sample size of 384 subjects was needed to ensure sufficient statistical power.

### Data collection

A self-administered structured questionnaire was used to collect the participants’ information. The survey was taken from the National Institutes of Health and National Cancer Institutes (NHANES) and was modified to fit the Saudi diet and objective of the study [12]. Content validity was achieved by two clinical dietitians and a public health expert. Face validity was achieved by a translator. For the purpose of ensuring the validity of the survey, a pilot study was conducted with a sample of 20 participants. The pilot study was conducted to assess the clarity of the questions in the survey and to identify any potential issues with the survey design. The feedback from the pilot study participants was collected and analysed to make any necessary adjustments to the survey before it was distributed to a larger population. The pilot study was conducted using a convenience sample of 20 participants who were selected based on their availability and willingness to participate. The participants were asked to complete the survey and provide feedback on the clarity of the questions and the overall survey design. The feedback was collected through a survey response form or an in-person interview. The feedback from the pilot study participants was carefully analysed, and any necessary adjustments were made to the survey before distributing it to a larger population. The adjustments made were aimed at improving the clarity of the questions, minimizing any potential biases, and ensuring that the survey was easy to understand and complete for all participants.

The questionnaire included three main sections. The first section covered questions related to added sugar intake in drinks. The second section covered added sugar intake in food, and the last section included demographic information, including age, gender, marital status, nationality, employment status, income, education, residential location (province), and chronic medical conditions.

### Dietary assessment

Due to the unavailability of some specific food items in NHANES dietary survey, we modified our food/drink diary survey to estimate the added sugar intake using a systemic methodology [13]. We determined added sugar in our modified dietary survey for added food items using the definition of added sugars as “sugars that are added to foods as an ingredient during preparation, processing, or at the table; added sugars do not include naturally occurring sugars such as lactose present in milk and fructose present in whole or cut fruit and 100% fruit juice” [14]. The online dietary survey was conducted to collect information about serving sizes (gram, and teaspoon), times (times, days, week and month) and type of intake (food/drink) recorded in the food diary. The participants’ dietary intakes were estimated by adding up the reported intakes of the modified food diary after the conversions and unifying of all measurements to gram/day. And then we multiplied the precalculated content data of sugar per gram for each food and drink item of the modified list of food, by the participants’ reported added sugar intakes gram/day. The final step was to add up all added sugar intakes gram/day for each participant in the collected data and divided by the number of food and drink items separately.

### Added sugar variables

The food diary survey consisted of two food/drink sections and a demographic section. The drink section included the following items: sweetened tea and coffee, sweetened fresh juice/canned juice/ cocktails, flavouring syrups, soft drinks, including Cola, Pepsi, 7up, Merinda, Mountain Dew, non-diet energy drinks, including, red bull, code red, Monster, Powerhorse, and Bison, flavored milk or flavoured Laban (skimmed milk). And the food section included the following items: chocolate, granola or an energy bar, cake, cheesecake, brownies, cookies, donuts, pie, muffin, cinnamon rolls, Danish or other sweets, Arabic sweets, sweetened cereal, oatmeal, porridge, ice cream or frozen yoghurt, jam, pomegranate molasses or any molasses with food, Ketchup, honey mustard, barbeque sauce, candy, sweetened popcorn, nuts with chocolates, sweetened dried fruits and vegetables.

### Ethical considerations

Participation in the study was voluntary. Completing the survey was construed as consent to participate. Participants were able to withdraw at any time. All questions were anonymized to preserve privacy. All information was kept with the authors only and was used for research purposes only. Data was stored electronically in an encrypted file to ensure the security and privacy of information. This study was carried out with the approval from the Institutional Review Board at Alfaisal University.

### Scoring

Scoring sheet for added sugar intake survey

Answer choices categorization/scoring:

Never = 0
1 time per month of less = 0.03
2–3 times per month = 0.08
1–2 times per week = 0.21
3–6 times per week = 0.64
1 time per day = 1
2–3 times per day = 2.5
More than 3 times a day = 3.5
Multiply by average number of sugar grams from the updated excel sheet
To come up with the total sugar intake per day

### Statistical analysis

Descriptive statistics were used to examine the differences in demographic characteristics among each added sugar intake, t-test statistics were used. To examine significant added sugar intake demographic predictors, we performed multivariate analyses using the least squares approach to general linear modeling adjusted for potential confounders. Assumptions of a linear function of the independent coefficients, error, and assumptions of normality and homogeneity of variance component were examined. A p-value < 0.05 was considered statistically significant for the multivariate analyses. All statistics were performed using SAS 9.4.

To ensure that numeric variables in the data were normally distributed, Q-Q plots and Shapiro-Wilk tests were employed [15]. Following the normality test, we employed parametric tests for all analyses. Univariate analyses using t-tests were conducted to assess differences in demographic characteristics across different levels of added sugar intake. To identify significant predictors of added sugar intake demographics, we utilized multivariate analyses using the least squares approach in general linear modelling, adjusted for potential confounders such as demographics. We further generated boxplots for visual illustrations. We evaluated assumptions of a linear function of the independent coefficients, error, normality, and homogeneity of variance component [16]. A p-value < 0.05 was considered statistically significant for the multivariate analyses. All statistical analyses were performed using SAS 9.4 software.

## Results

**Fig 1:**
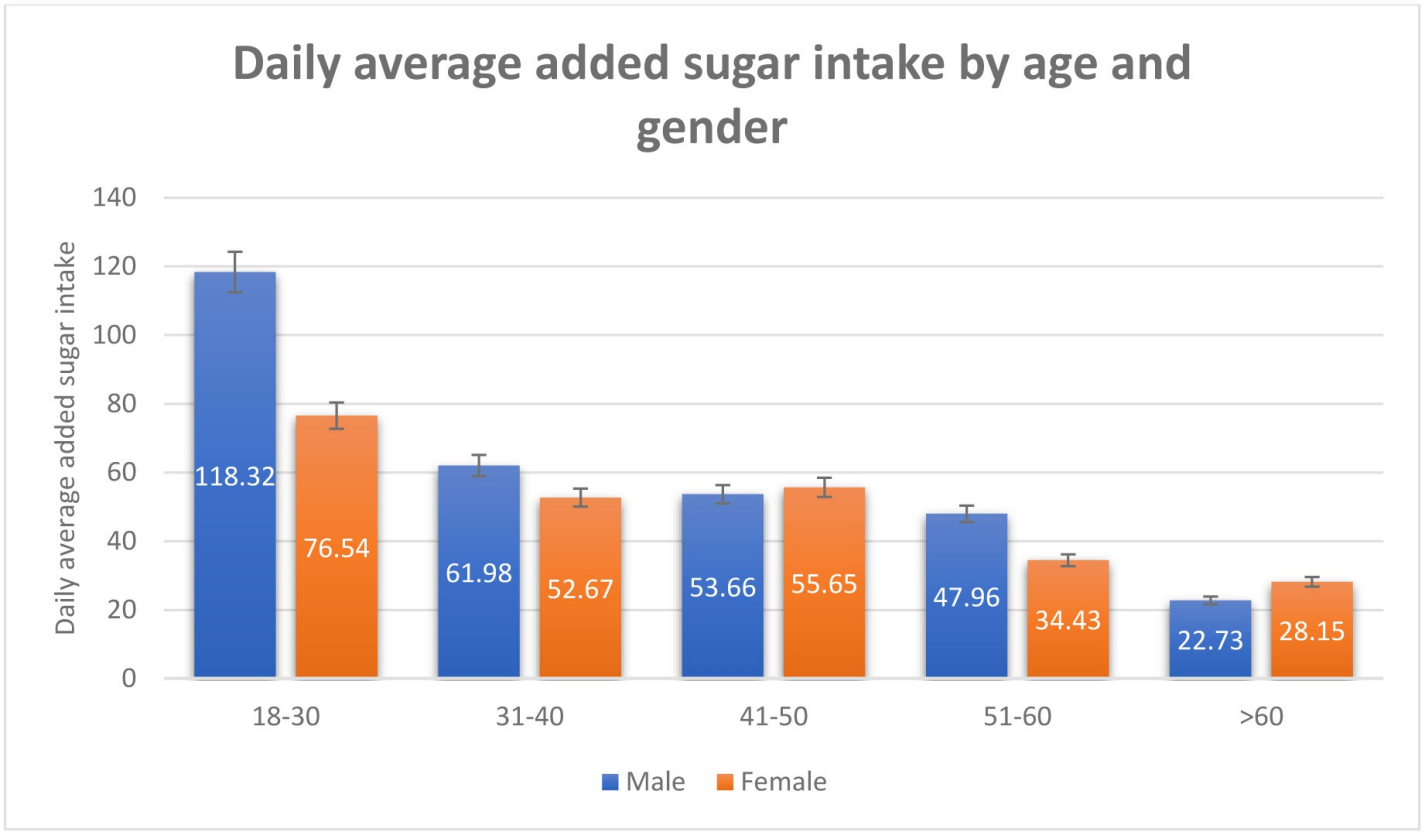
Daily average added sugar intake by age and gender.

**Fig 2:**
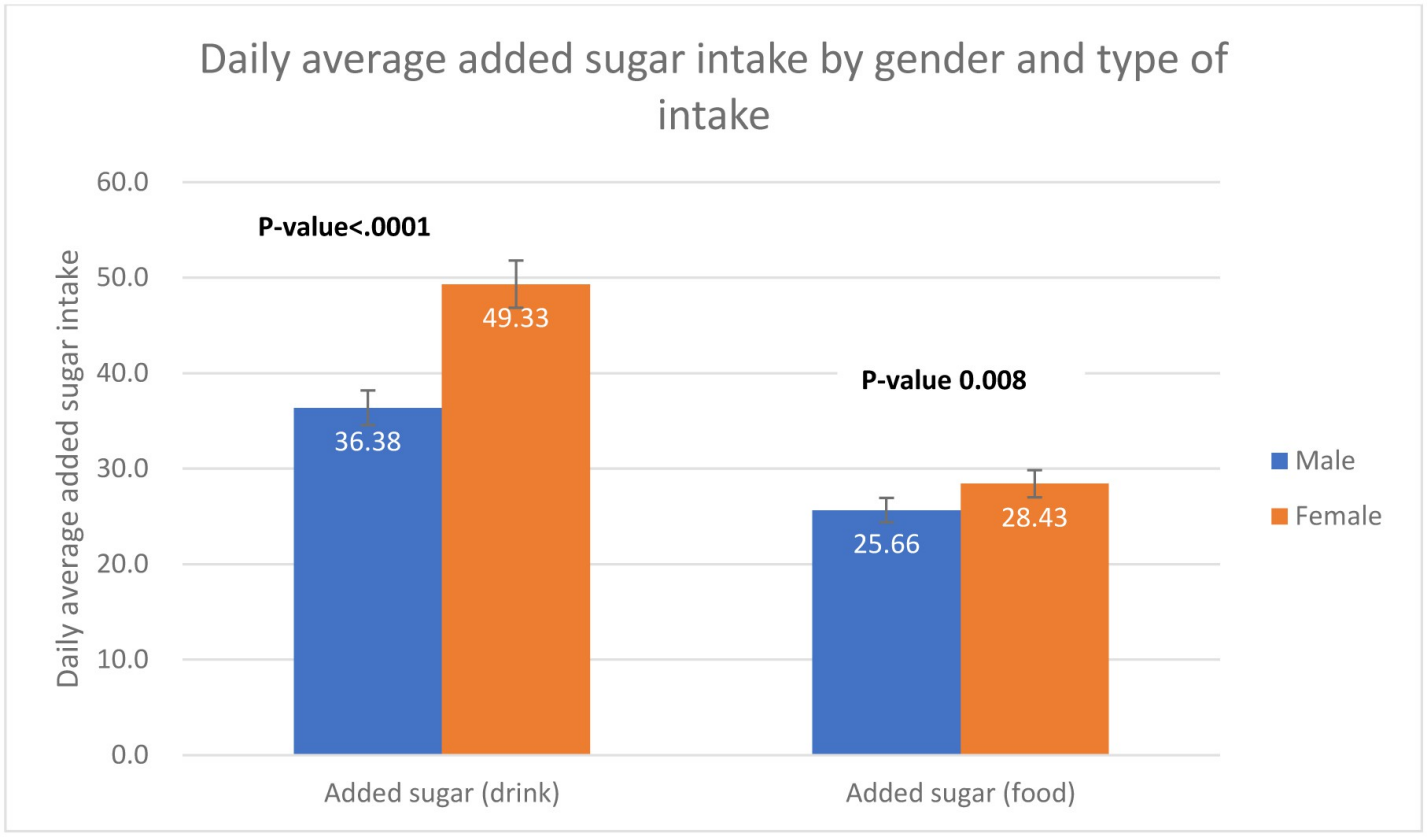
Daily average added sugar intake by gender and type of intake (drinks vs. food).

**Fig 3:**
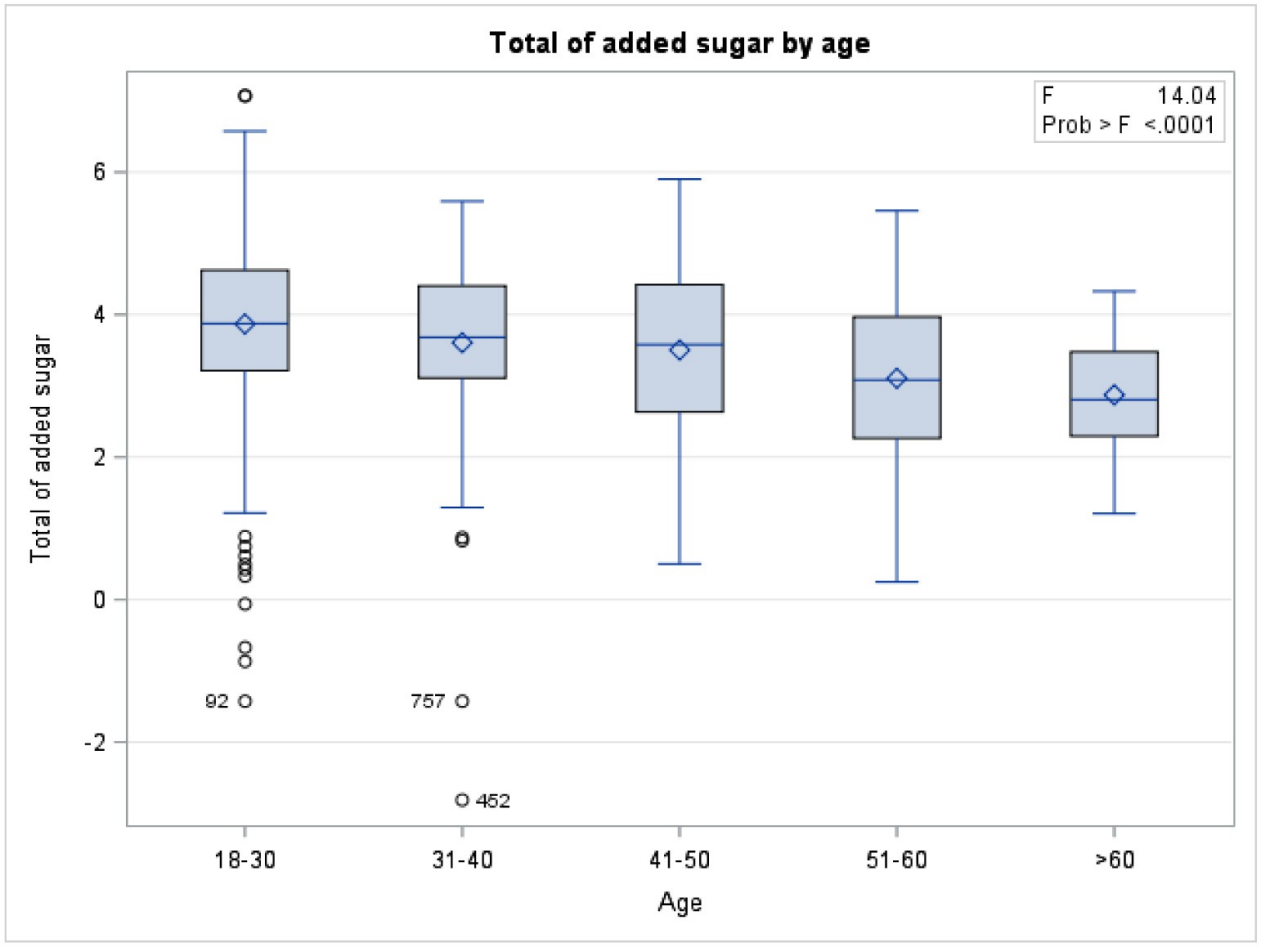
Added sugar intake by age.

**Fig 4:**
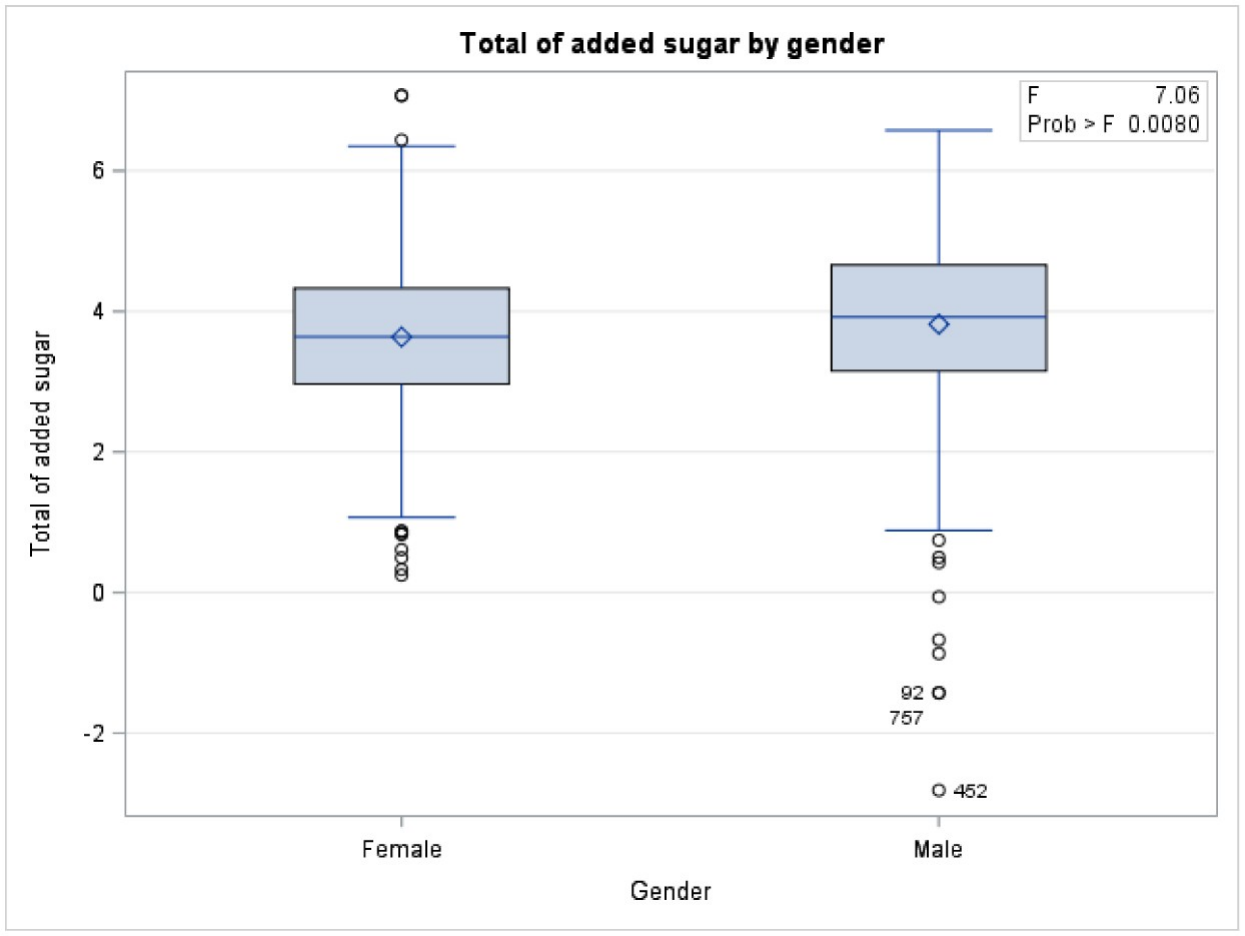
Added sugar intake by gender.

A total of 1163 respondents included in the study. More than half of the population were aged 18–30 years old (61.34%), female (59.79%), single (59.62%), non-obese (76.2%), reported no comorbidity (78.95%), non- employed (58.42%), Saudi national (63.4%), and residing in the central region of Saudi Arabia (51.89%) (Table 1). For both males and females, the means of added sugar intake have decreased as the age increased (Fig 1). There was a significant difference in means of overall added sugar intake across genders for age group 18–30 and age group >60 (Fig 1). Equivalently, there was a statistically significant difference in means of added sugar intake (Food) across gender (P-value 0.008), females tended to consume more added sugar through food compared to males (Fig 2). In the contrary, males consume more added sugar through drink compared to females (p-value <0.0001) (Fig 2). An interaction of age and gender showed a statistically significant difference in means of added sugar intake across gender with different age group (Table 2). The means of added sugar intake was significantly different in both male and female youth and younger older adults age groups (18–30), (31–40), and (41–50) compared to (>60) age group (Table 2). In terms of added sugar intake, compared to age group >60, there was a statistically significant difference in means of added sugar intake across age groups 18–30, 31–40, and 41–50 (mean SE 86.09 ± 0.25, P value 0.0001; mean SE 57.98±0.24, P value 0.005; mean SE 54.91 ± 0.25, P value 0.017, respectively) (Table 1 and Fig 3). Age group 18–30 tended to have the highest mean of added sugar intake compared to other age group. Same pattern was observed across gender and body mass index. There was a statistically significant difference in means of added sugar intake between male and female, obese versus non-obese respondents, and respondents reported comorbidity (mean SE 66.198 ±0.07, P value 0.02; mean SE 71.59 ± 0.08, P value 0.053; mean SE 75.19 ± 0.08, P value 0.0007, respectively) (Table 1 and Fig 4). There was no statistically significant difference in added sugar consumption among different regions. The highest consumption was in the northern region (123.71 g/day), followed by the southern region (98.52 g/day), then the western region (86.14 g/day), and lastly, the central/Riyadh region and eastern region (66.95 and 62.02 g/day, respectively).

**Table 1:** Difference in means of daily average added sugar intake gram/day.

Total	Sample n (n%) ^2^	Total added sugar (gram/day) consumption overall means	*P* value ^1^
	N = 1162	Total mean = 73.04 Mean ± Standard error	
**Age**			
18–30	714 (61.34)	86.09 ± 0.25	0.0001
31–40	238 (20.45)	57.98±0.24	0.005
41–50	109 (9.36)	54.91 ± 0.25	0.017
51–60	79 (6.79)	39.86 ±0.26	0.33
>60	24 (2.06)	25.44	**Reference**
**Gender**			
Male	468 (40.21)	66.198 ±0.07	0.022
Female	696 (59.79)	83.22	**Reference**
**Marital status**			
Married	430 (36.94)	56.41 ± 0.56	0.48
Single	694 (59.62)	83.77 ± 0.57	0.52
Divorced	36 (3.09)	70.33 ± 0.59	0.22
Widowed	4 (0.34)	24.37	**Reference**
**Body Mass Index**			
Underweight	95 (8.16)	118.41±0.12	Reference
Normal	516 (44.33)	70.73±0.14	0.005
Overweight	313 (26.89)	55.55±0.13	0.0001
Obese	240 (20.62)	82.85	0.21
**Comorbidity**			
Yes	245 (21.05)	64.98	**Reference**
No	919 (78.95)	75.19 ± 0.08	0.007
**Are you employed?**			
Yes	484 (41.58)	76.22	**Reference**
No	680 (58.42)	68.57 ±0.1	0.057
**What is your nationality?**			
Saudi	738 (63.4)	58.59	**Reference**
Non- Saudi	426 (36.6)	81.38 ± 0.071	0.028
**What is your monthly income?**			
9,999 Saudi Riyal or less	233 (20.02)	82.03 ± 0.1	0.55
10,000–19,999 Saudi Riyal	145 (12.46)	64.17 ± 0.12	0.52
More than 20,000 Saudi Riyal	150 (12.89)	49.97 ± 0.13	0.23
I prefer not to answer	240 (20.62)	79.34	**Reference**
I don’t have monthly income	396 (34.02)	75.91 ±0.1	0.63
**What is the level of your education?**			
High school or less	386 (33.16)	77.73 ± 0.11	0.21
Undergraduate degree	548 (47.08)	79.81 ± 0.097	0.025
Graduate degree	230 (19.76)	49.04	**Reference**
**Region**			
Central region	604 (51.89)	66.95 ± 0.12	0.17
Western region	100 (8.59)	86.13 ± 0.12	0.12
North region	88 (7.56)	123.71	**Reference**
South region	55 (4.73)	98.52 ±0.16	0.26
Eastern region	317 (27.23)	62.02 ±0.18	0.86

^1^ General linear model p-value

^2^ n(%) sample size and percentage

**Table 2:** Comparison of mean overall added sugar intake across interaction of age and gender.

Age*Gender	Estimate	Standard error	P-value ^1^
**Female 18–30**	0.977	0.32	0.002
**Female 31–40**	0.78	0.33	0.019
**Female 41–50**	0.75	0.35	0.03
**Female 51–60**	0.16	0.35	0.65
**Female >60**	0.17	0.45	0.71
**Male 18–30**	1.23	0.32	0.0002
**Male 31–40**	0.85	0.34	0.012
**Male 41–50**	0.64	0.36	0.081
**Male 51–60**	0.61	0.38	0.11
**Male >60**	Reference	Reference	Reference

^1^ General linear model p-value

## Discussion

High daily sugar intake is correlated with obesity, chronic diseases, and cancer; therefore, the current study estimated the prevalence of sugar consumption among Saudi and non-Saudi residents in Saudi Arabia. Due to the excess sugar intake, several studies have investigated sugar intake among different populations. The recommended sugar intake by AHA is 24g/day and 36g/day for women and men, respectively. Our study has shown an overall added sugar intake average of 73g/ day (83g/day and 66g/ day for men and women, respectively) which is more than double the recommended amounts. A cross-sectional study conducted among 190 healthy female undergraduate students at Taibah University, Madinah, Saudi Arabia, indicated that 45.8% of students consumed sweets daily, 15.8% consumed fruit drinks daily, and 8.42% of the students consumed carbonated soft drinks daily [17]. Another cross-sectional study among 414 undergraduate students at King Faisal University in Al-Ahssa, Saudi Arabia, revealed that 40% of students reported consuming soft drinks once daily, and almost one-third of them (27.5%) two or more daily. The majority of them consume soft drinks weekly (69.6%) then, energy drinks (40.6%), and sports drinks (25.6%) [18]. Our study shows an antiparallel relationship between age and sugar consumption, where individuals o aged 60 years old illustrate reasonable sugar intake compared to 18–30 years old. The gradual decrease in sugar intake according to age group presumably reflects the awareness level. Studies demonstrated higher sugar-based diet consumption in young individuals. In fact, a study computed in Malaysia showed that the estimated mean intake of total sugar per day among age groups 18 to 29 was higher than in older age groups [19]. Another similar study computed—sweetened beverages intake among adults, which showed that those aged 18–24 had 2.3 times higher SSB consumption than older age groups [20].

A study suggested male undergraduate students consume more sugar than female undergraduate students [21]. Also, another study demonstrated that men showed higher sugar intake than women; however, in relative terms, this observation was the opposite, which reflects the greater energy intake by men inherent in their constitution that demands, more significant energy needs. In contrast, higher relative values in women demonstrate that they are consuming a more nutritionally unbalanced diet, at least as far as sugar intake is concerned [22]. This finding of higher overall added sugar consumption among women is consistent with our present data, which could be explained by our cultural practices where women are more likely to be exposed to desserts which are always offeredduring family and friends gatherings. Furthermore, when looking into foods and drinks separately, previous local studies have shown that the daily consumption of sugary foods was more significant among females. On the other hand, males were found to consume significantly overall quantities of sugary drinks compared to females (P = 0.000) [23]. Additionally, another cross-sectional study conducted in Lebanon showed that females reported significantly higher daily consumption of all sugary foods than males did [24]. Those findings are consistent with our study findings which showed higher consumption of sugary foods among females and higher consumption of sugary drinks among males. Two factors recently proposed to explain the increased consumption of sugary foods among females are hormones and stress [25]. The finding of higher sugary drinks consumption among males is not surprising given that males are traditionally perceived as more active and mobile in Eastern countries and more likely to do sports and thus drink more of the sugary energy drinks, sports drinks, and sweetened bottled waters.

A study done in the US showed that BMI increased concurrently with added-sugar intake in both genders and all age and weight groups [26]. Another study indicated that liquid but not solid added sugars were positively associated with higher BMI [27]. However, those findings are inconsistent with our study findings which showed an inverse relationship between BMI and sugar consumption, in which underweight individuals had the highest intake of added sugars, followed by obese, normal, and lastly, the overweight. This pattern indicates that underweight individuals take their freedom with consuming added sugars as they seem not to see the effects of that consumption on their bodies. However, obese individuals seem to be slightly more cautious about their consumption, reflecting their will to control and lower their BMI and weight. Sugar consumption is linked to many comorbidities such as obesity, heart diseases, diabetes mellitus, and cancer. For instance, a cohort study showed that total sugar intake was associated with higher overall cancer risk including prostate and breast cancer [28].

Similarly, a meta-analysis has shown a clear link between sugar-sweetened beverages (SSB) consumption and the risk of metabolic syndrome and type 2 diabetes [29]. On the contrary, our findings revealed a startling lack of connection between sugar intake and comorbidities. This might be because most individuals were young when comorbidities were less prone to appear.

It has been suggested that financial income influences sugar intake; according to estimates, this element does not significantly affect sugar intake among Saudi nationals [30]. Our study findings were consistent with previous studies regarding the relation with income. One suggestion that could justify this is that deserts and sweet beverages are a cultural necessity, irrespective of the person’s financial situation. However, our study showed that employed people consumed more sugar than the unemployed. This can be explained by the fact that employed individuals are more vulnerable to unhealthy diets due to their busy daily schedules. It could be related to the high energy requirements needed for workplace productivity and easier access to sugar.

Our present study has shown that non-Saudis have a higher sugar intake than Saudis. This finding could be due to the fact Saudi Arabia hosts more than 10 million non-Saudis, mostly of Indian, Syrian, and Pakistani nationalities [31]. Those nations are known to consume higher sugar due to their cultural food cuisines (e.g., Kunafa, Basbosa, Baklava, Gulab jamum, Jalebi, etc.). A 24-hr food intake recall and frequency of sugar intake showed that the total sugar consumption was significantly lower in the educated group. Similarly, our study results were consistent with the data. Therefore, it is suggested that education level influences an individual’s diet choice and thus their sugar intake [32].

The study does have certain drawbacks. The study’s cross-sectional design restricts any causal findings, recall and selection bias are significant constraints. Another limitation is the convenience sampling. However, this study sheds light on the added sugar consumption in Saudi Arabia, which is very limited in the literature. The sample size is relatively big.

## Conclusion

The total intake of added sugar is extremely high in Saudi Arabia. Poor dietary habits lead to many negative health consequences, including obesity and diabetes. Healthcare providers and public health officials are highly encouraged to shed light on added sugar consumption and promote healthy diet patterns. The Saudi population is recommended to abide by the added sugar dietary recommendations to avoid chronic medical conditions. Future research is needed to generalize this study and better understand added sugar consumption from various health perspectives.

## Supporting information

S1 Appendix Scoring sheet for added sugar intake survey. (DOCX)
