# Supplementary material for: Added sugar intake among the saudi population
Source: PLoS One. 2023 Sep 8;18(9):e0291136. doi: 10.1371/journal.pone.0291136 (PMC10490978; doi:10.1371/journal.pone.0291136)
Supplement: S1 Appendix — (DOCX) [file pone.0291136.s001.docx]

Appendix

Scoring:

Scoring sheet for added sugar intake survey

Answer choices categorization/scoring:

Never= 0

1 time per month of less= 0.03

2-3 times per month= 0.08

1-2 times per week= 0.21

3-6 times per week= 0.64

1 time per day= 1

2-3 times per day= 2.5

More than 3 times a day= 3.5

Multiply by average number of sugar grams from the updated excel sheet

To come up with the total sugar intake per day

Start here:

Multiply questions 1*2

Q1

Never= 0

1 time per month of less= 0.03

2-3 times per month= 0.08

1-2 times per week= 0.21

3-6 times per week= 0.64

1 time per day= 1

2-3 times per day= 2.5

More than 3 times a day= 3.5

Q2:

1 cube= 2 grams

3 cubes= 10 grams

>3 cubes= 12

Q3: *10 grams of sugar

Never= 0

1 time per month of less= 0.03

2-3 times per month= 0.08

1-2 times per week= 0.21

3-6 times per week= 0.64

1 time per day= 1

2-3 times per day= 2.5

More than 3 times a day= 3.5

Q4: *30

Never= 0

1 time per month of less= 0.03

2-3 times per month= 0.08

1-2 times per week= 0.21

3-6 times per week= 0.64

1 time per day= 1

2-3 times per day= 2.5

More than 3 times a day= 3.5

Q5: *17

Never= 0

1 time per month of less= 0.03

2-3 times per month= 0.08

1-2 times per week= 0.21

3-6 times per week= 0.64

1 time per day= 1

2-3 times per day= 2.5

More than 3 times a day= 3.5

Q6: *6

Never= 0

1 time per month of less= 0.03

2-3 times per month= 0.08

1-2 times per week= 0.21

3-6 times per week= 0.64

1 time per day= 1

2-3 times per day= 2.5

More than 3 times a day= 3.5

Q7: *39

Never= 0

1 time per month of less= 0.03

2-3 times per month= 0.08

1-2 times per week= 0.21

3-6 times per week= 0.64

1 time per day= 1

2-3 times per day= 2.5

More than 3 times a day= 3.5

Q8: *38

Never= 0

1 time per month of less= 0.03

2-3 times per month= 0.08

1-2 times per week= 0.21

3-6 times per week= 0.64

1 time per day= 1

2-3 times per day= 2.5

More than 3 times a day= 3.5

Q9: *23

Never= 0

1 time per month of less= 0.03

2-3 times per month= 0.08

1-2 times per week= 0.21

3-6 times per week= 0.64

1 time per day= 1

2-3 times per day= 2.5

More than 3 times a day= 3.5

Food

Q1: *22

Never= 0

1 time per month of less= 0.03

2-3 times per month= 0.08

1-2 times per week= 0.21

3-6 times per week= 0.64

1 time per day= 1

2-3 times per day= 2.5

More than 3 times a day= 3.5

Q2: *10

Never= 0

1 time per month of less= 0.03

2-3 times per month= 0.08

1-2 times per week= 0.21

3-6 times per week= 0.64

1 time per day= 1

2-3 times per day= 2.5

More than 3 times a day= 3.5

Q3: *16

Never= 0

1 time per month of less= 0.03

2-3 times per month= 0.08

1-2 times per week= 0.21

3-6 times per week= 0.64

1 time per day= 1

2-3 times per day= 2.5

More than 3 times a day= 3.5

Q4: *23

Never= 0

1 time per month of less= 0.03

2-3 times per month= 0.08

1-2 times per week= 0.21

3-6 times per week= 0.64

1 time per day= 1

2-3 times per day= 2.5

More than 3 times a day= 3.5

Q5: *12

Never= 0

1 time per month of less= 0.03

2-3 times per month= 0.08

1-2 times per week= 0.21

3-6 times per week= 0.64

1 time per day= 1

2-3 times per day= 2.5

More than 3 times a day= 3.5

Q6: *13

Never= 0

1 time per month of less= 0.03

2-3 times per month= 0.08

1-2 times per week= 0.21

3-6 times per week= 0.64

1 time per day= 1

2-3 times per day= 2.5

More than 3 times a day= 3.5

Q7: *16

Never= 0

1 time per month of less= 0.03

2-3 times per month= 0.08

1-2 times per week= 0.21

3-6 times per week= 0.64

1 time per day= 1

2-3 times per day= 2.5

More than 3 times a day= 3.5

Q8: *3

Never= 0

1 time per month of less= 0.03

2-3 times per month= 0.08

1-2 times per week= 0.21

3-6 times per week= 0.64

1 time per day= 1

2-3 times per day= 2.5

More than 3 times a day= 3.5

Q9: *2

Never= 0

1 time per month of less= 0.03

2-3 times per month= 0.08

1-2 times per week= 0.21

3-6 times per week= 0.64

1 time per day= 1

2-3 times per day= 2.5

More than 3 times a day= 3.5

Q10: *16

Never= 0

1 time per month of less= 0.03

2-3 times per month= 0.08

1-2 times per week= 0.21

3-6 times per week= 0.64

1 time per day= 1

2-3 times per day= 2.5

More than 3 times a day= 3.5

Q11: *8

Never= 0

1 time per month of less= 0.03

2-3 times per month= 0.08

1-2 times per week= 0.21

3-6 times per week= 0.64

1 time per day= 1

2-3 times per day= 2.5

More than 3 times a day= 3.5
